# Assessment on Available Livestock Feed Resources and Feeding System in Korahey Zone, Somali Region, Ethiopia

**DOI:** 10.1002/vms3.71141

**Published:** 2026-08-05

**Authors:** Wondimagegn Tadesse, Kibebew Babege, Mahamed Abdi

**Affiliations:** ^1^ Department of Animal Science College of Dry‐Land Agriculture Kebri Dehar University Kebri Dehar Ethiopia

**Keywords:** constraints, dry matter, feed source, feeding system

## Abstract

**Objective:**

This study aimed to assess livestock feed resources, feeding systems, feed availability and major feed‐related constraints in the Korahey Zone of the Somali Region, Ethiopia.

**Materials and Methods:**

A cross‐sectional survey was conducted in four purposively selected districts of the Korahey Zone. Three kebeles (the smallest administrative unit in Ethiopia) were selected from each district, and 240 livestock‐owning households were purposively sampled. Data on livestock population, landholding size, feed resources, feeding systems and feed‐related constraints were collected using a semi‐structured questionnaire. The data were analysed using the Statistical Package for the Social Sciences (SPSS) version 20. Feed‐related constraints were prioritized using a ranking index, while dry matter (DM) supply and livestock maintenance requirements were estimated to determine the feed balance.

**Results:**

Camels and goats were the dominant livestock species in the study area. Natural pasture was the principal feed resource (75%), and 90% of respondents practised free grazing on natural pasture. The estimated annual feed supply from natural pasture was 2196 tons DM, whereas the annual livestock maintenance requirement was 4023.68 tons DM, indicating that available feed met only 54.6% of the maintenance requirement. The major feed resources included natural pasture, crop residues, improved forages, household food leftovers, maize and sorghum. The main constraints affecting feed availability were drought, grazing land shortages, limited availability of improved forage seeds, feed shortages, invasive plant species, inadequate extension services and land‐use conflicts.

**Conclusion:**

Livestock production in the Korahey Zone is constrained by insufficient feed resources, resulting in a substantial feed deficit. Improving livestock productivity requires rangeland rehabilitation, expansion of improved forage production, better feed resource management, strengthened extension services and further research to develop sustainable feeding strategies for the area.

## Graphical abstract

Livestock feeding in the Korahey Zone, Somali Region, Ethiopia, is predominantly based on free grazing of natural pasture (75%), with camels and goats being the dominant livestock species. The annual feed supply from natural pasture (2196 tDM) satisfies only 54.6% of the livestock dry matter requirement (4023.68 tDM), resulting in a substantial negative feed balance. Drought, grazing land shortages, lack of quality forage seeds, feed shortages, invasive species expansion, inadequate extension services and land‐use conflicts were identified as the major constraints. These findings highlight the urgent need for rangeland rehabilitation, improved forage development, strengthened extension services and sustainable feed‐resource interventions to enhance livestock productivity in the area

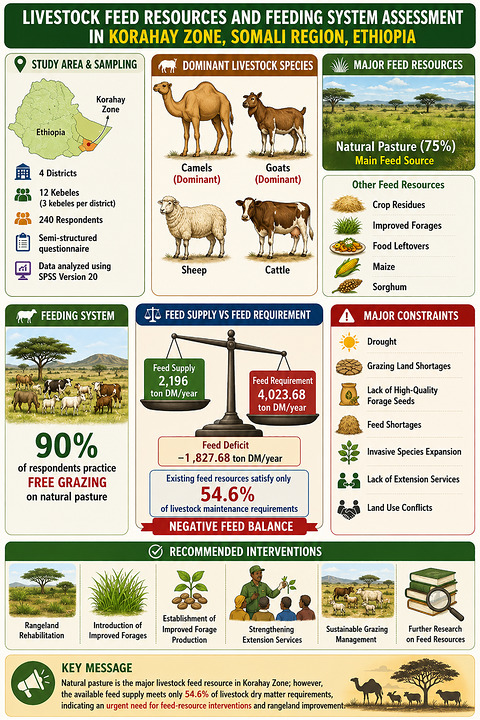

**Abbreviations**
CSA: Central Statistical Agency
DM: dry matter
FAO: Food and Agriculture Organization
HH: household
tDM: ton dry matter

## 1 Introduction

Livestock feed resources in Ethiopia are obtained mainly from natural grazing and browsing, crop residues, improved forages, agro‐industrial byproducts and vegetable and fruit refuse, of which the first two are quantitatively significant (Tolera et al. 2012). The contribution of these feed resources depends on agroecology, the type of crop produced, accessibility and the production system (Ahmed et al. 2012). Natural pastures are characterized by seasonal fluctuations in total dry matter (DM) production and nutritional quality because of the distinct seasonal variation in plant growth in relation to the annual rainfall pattern. Crop residues are fibrous and high in lignin content, which limits their feeding value (McDonald et al. 2002; Adugna 2009) and results in a low content of essential nutrients (proteins, energy, minerals and vitamins) and low digestibility and intake. The poor nutritive value of grasses and their lower degradability result in low intake and utilization, thereby reducing the performance of these animals (Sisay 2006).

The feeding of livestock in different places differs depending on forage availability, climatic conditions, season of the year and type of animal (Teklu et al. 2011). A livestock feeding calendar is an essential livestock management practice to use the available feed resources efficiently, to supply livestock with sufficient and high‐quality feed and to overcome feed shortages. The livestock feeding calendar varies depending on the availability of feed resources in different months of the year (Mengistu 2003). The feeding systems in the country include communal or private natural grazing and browsing, cut‐and‐carry feeding, hay and crop residues.

In the arid and semi‐arid lowlands of the Somali Regional State, the livestock feed situation is even more challenging than in the Ethiopian highlands, as pastoral and agro‐pastoral production systems rely heavily on seasonal rangelands and browsing species. Feed availability in these areas is highly influenced by erratic rainfall patterns, recurrent droughts and degradation of grazing lands, resulting in severe seasonal fluctuations in both feed quantity and quality. Consequently, livestock producers in the Somali region face persistent feed shortages, particularly during the dry season, which adversely affects livestock productivity and household (HH) livelihoods (Adugna 2009; Yisehak et al. 2014; T. Solomon et al. 2018).

According to Zewdie et al. (2010), assessment of the quantity and quality of available feed resources in relation to livestock requirements has not yet been well addressed in most livestock production areas of the country. Feeds are either unavailable or in an insufficient quantity due to fluctuating weather conditions (climate change) in the Korahey Zone. Understanding the types of available livestock feed resources and their balance, as well as designing appropriate livestock feeding strategies and feeding systems, are important for improving the productivity of livestock and providing appropriate knowledge to smallholder farmers. Therefore, this study attempts to identify the types of livestock feed sources, their feeding system practices, and the balance between the available feed and nutrient requirements of livestock in the Korahey Zone of the Somali Regional State, Ethiopia. This will help generate baseline information on the available feed sources, feeding system and feed supply balance for communities rearing livestock, particularly browsers, and for policymakers, researchers, stakeholders and the Bureau of Livestock. Thus, this study aimed to assess available livestock feed resources and the existing feeding system in the Korahey Zone, Somali Region, Ethiopia.

## 2 Materials and Methodology

### 2.1 Description of the Study Area

The Somali Region is one of the largest regions in Ethiopia. The region comprises eleven administrative zones. The climate is mostly arid/semi‐arid in lowland areas, with altitude generally ranging from about 300–1600 m above sea level, with most areas lie below 1000 m, and the annual rainfall ranges from 150–600 mm per year.

**FIGURE 1:**
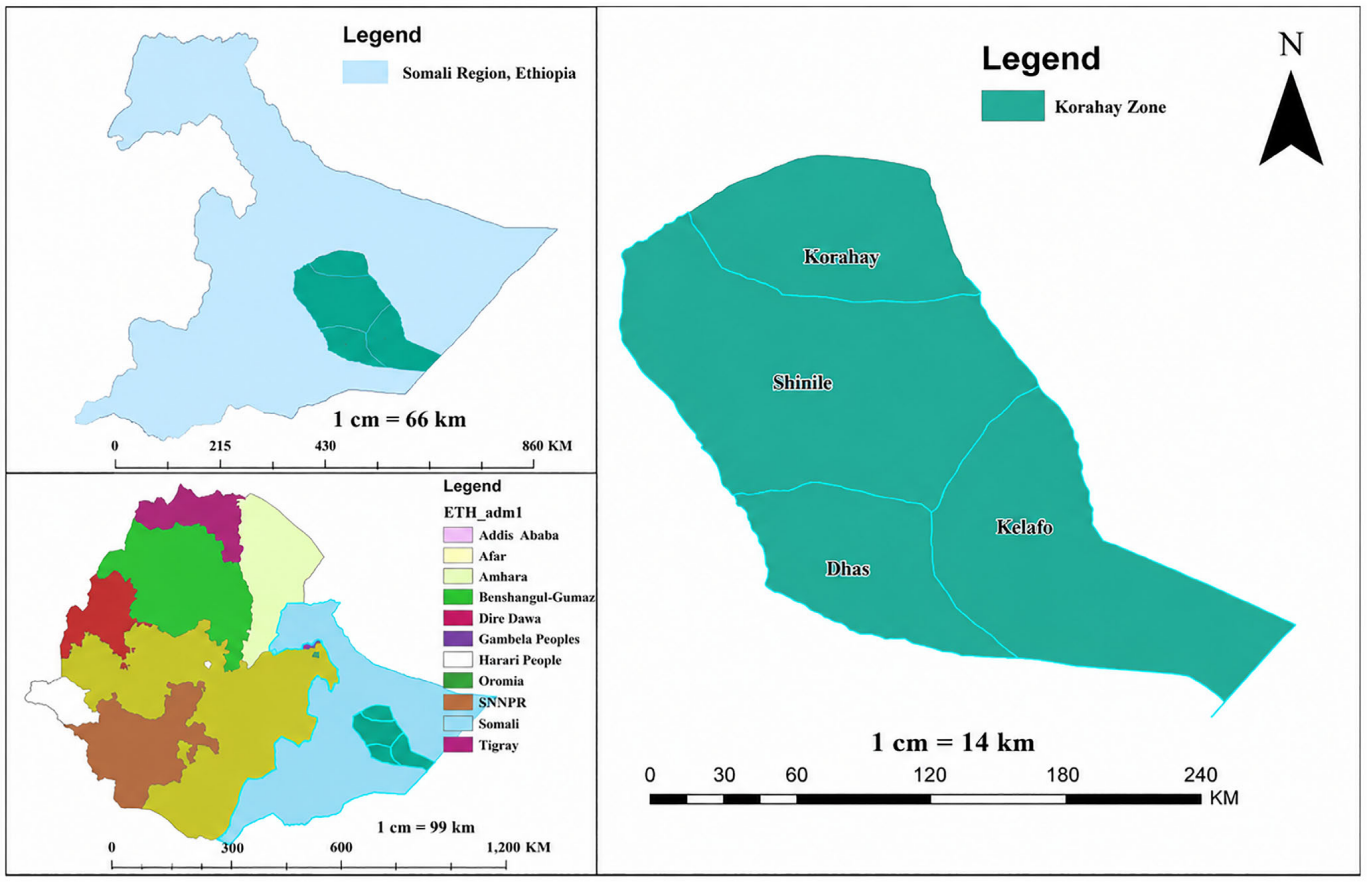
Map of the study area. The figure shows the geographical location of the Korahey Zone within the Somali Region of Ethiopia, with district‐level administrative boundaries and the position of the study area within the national context.

Korahey Zone is one of the administrative zones from 11 zones, and it receives ‘Gu’ rains (main season) from mid‐April to the end of June and secondary rains known as ‘Deyr’ from early October to late December. Shilabo, Boodele, Lass‐Dankhare and Kabridahar are districts located in the Korahey Zone of the Somali Region. Kebri Dehar town has a latitude and longitude of 6°44′N 44°16′E and an elevation of 1609 m above sea level (Figure 1).

Study district mean annual temperatures range from 20.75°C to 31.25°C, while annual rainfall varies between 295 and 595.6 mm (Korahey Zone Agricultural Office, 2024). The months of June, July, August and September are considered as wet season, while other months are dry. However, there was change from year to year.

### 2.2 Study Design

A cross‐sectional descriptive survey design was employed to assess livestock species composition, feed resource availability and potential of feed resources (grasses, forbs, fodder trees and crop production), feeding systems, and landholding characteristics of livestock‐keeping HHs in the Korahey Zone of the Somali Regional State, Ethiopia.

### 2.3 Sampling Method and Sample Size

Both purposive and random sampling methods were employed to select samples of HHs. The four districts (Shilabo, Boodele, Lass‐Dankhare and Kabridahar) were selected purposively on the basis of their livestock population and drought‐prone areas; a geographic area that frequently experiences drought, where rainfall is low, irregular, or unreliable, leading to water shortages, poor pasture growth and reduced crop and livestock productivity. On the basis of the above criteria, the populations (owning livestock) were purposively selected. A cross‐sectional survey was subsequently conducted prior to the main survey to identify the distribution of livestock, and three kebeles (the smallest administrative unit in Ethiopia) with greater potential for livestock were selected from each districts. On the basis of the cross‐sectional survey and secondary information gathered from the zonal and district levels of agricultural and pastoralist offices, 12 kebeles were selected. Accordingly, a total of 240 HH heads (sample size) were selected (20 HHs from each kebele) randomly as a result of homogeneity of production systems.

### 2.4 Data Collection Techniques and Data Sources

The assessment focused on five major areas: landholding capacity, major available feed resources, livestock species and herd size, feeding systems and primary constraints related to feed in the selected districts of the Korahey Zone.

Structured and semi‐structured questionnaires were developed to guide data collection. Primary and secondary data were obtained through focus group discussions with key informants and experts from the district livestock and fisheries offices. In addition to interviews and group discussions, direct observational studies were conducted to validate information collected through structured questionnaires and guided interviews.

Primary data were collected from smallholder farmers, while secondary or supplementary information, such as rainfall, temperature and landholding data, was obtained from the district livestock development and fisheries office, agricultural office, land and environmental protection office, kebele leaders and development agents. Interviews with HHs were conducted at the farmers’ homes, covering demographic characteristics, farming systems, herd size and composition, major available feed resources, feeding systems and constraints related to feed availability and utilization.

### 2.5 Estimation of Available Feed Resources and the DM

To assess the availability, sources and types of livestock feed in the district, data were collected from both primary and secondary sources. Secondary data on livestock populations and crop production potential were obtained by reviewing relevant documents, including annual reports, from district offices responsible for livestock production, agriculture and land management.

The annual quantity of feed DM from different land‐use types was estimated by multiplying the area under each land‐use category by its respective conversion factor. Conversion factors of 2.0, 0.5, 1.0, 5.0 and 0.7 tons DM/ha/year were applied for natural pasture, aftermath grazing, crop residues, improved forages and forestland, respectively (FAO 1984).

### 2.6 Estimation of the Livestock Population and DM Requirements

The total livestock population of the district and the interviewed HHs was converted to tropical livestock units (TLUs) as recommended by Jahnke (1982) for local animals. Therefore, the conversion factors for local oxen and bulls, cows, heifers and calves were 1, 0.7, 0.5, 0.2 and 27, respectively. For sheep and goats, conversion factors of 0.1 and 0.8 for horses, 0.5 for donkeys, 0.7 for mules and 0.01 for poultry were used. The DM requirement of an animal was calculated on the basis of the daily DM requirement of 250 kg of dual‐purpose tropical cattle (an equivalent of one TLU) for maintenance, which needs 6.25 kg/day/animal or 2281 kg/year/animal (Jahnke 1982).

### 2.7 Data Management and Statistical Analysis

The raw data were entered, edited, checked and managed via Microsoft Excel spreadsheet access. Data organization, summarization and analyses were performed after coding and rechecking in SPSS Statistics for Windows, Version 20.0. (Armonk, NY: IBM Corp.). A simple descriptive analysis was generated (means and proportions, counts and percentage summary statistics and tests of significance for differences in means and proportions were estimated at 95% confidence intervals, and a 5% (0.05) level of significance was considered the cutoff point).

## 3 Results

### 3.1 Livestock Composition

The major livestock species in the study area were goats, sheep, cattle, camels and poultry (Table 1). The average total livestock population per HH was 21.3 ± 2.43^b^, 20.45 ± 3.454^b^, 22.5 ± 2.532^a^ and 23.95 ± 2.802^a^ TLU in the Kebri Dehar, Lass‐Dankhare, Boodele and Shilabo districts, respectively (Table 1). The results revealed that camels and goats were the dominant livestock species in the sampled districts of the Korahey Zone.

**TABLE 1:** Livestock composition per household (TLU).

Livestock	Kebri Dehar	Lass‐Dankhare	Boodele	Shilabo	
	Mean ± SE	Mean ± SE	Mean ± SE	Mean ± SE	*p*‐value
Cattle	1.9 ± 1.29	3.2 ± 0.492	2.9 ± 1.219	2.55 ± 0.284	0.34
Sheep	3.1 ± 1.17	2 ± 0.53	3.3 ± 1.72	3.25 ± 1.274	0.40
Goat	3.5 ± 1.267^b^	4.05 ± 0.412^a^	4.3 ± 2.23^a^	3.75 ± 1.71^b^	0.022
Camel	12.8 ± 1.25^b^	11.2 ± 0.292^b^	12 ± 0.523^b^	14.4 ± 0.823^a^	0.056
Total	21.3 ± 2.43^b^	20.45 ± 3.454^b^	22.5 ± 2.53^a^	23.95 ± 2.802^a^	0.045

Abbreviation: SE, standard error. Means within the same row followed by different lowercase superscript letters (a, b) differ significantly among districts at P < 0.05, whereas means sharing the same lowercase superscript letter are not significantly different

### 3.2 Land Holding Capacity

The average landholding per HH in the surveyed districts of the Korahey Zone was limited and predominantly allocated to grazing. In Kebri Dehar, Lass‐Dankhare, Boodele and Shilabo, the mean grazing land per HH was 2.65 ± 0.79 ha, 2.81 ± 0.18 ha, 2.92 ± 0.69 ha and 3.40 ± 1.21 ha, respectively, with no significant differences among districts (*p* > 0.05). No cropland or crop aftermath grazing areas were reported by respondents, indicating a strong dependence on natural pasture for livestock feeding. This limited land availability underscores the pressure on grazing resources and the vulnerability of livestock production systems to feed shortages, particularly under recurrent drought conditions.

### 3.3 Available Feed Resources

**FIGURE 2:**
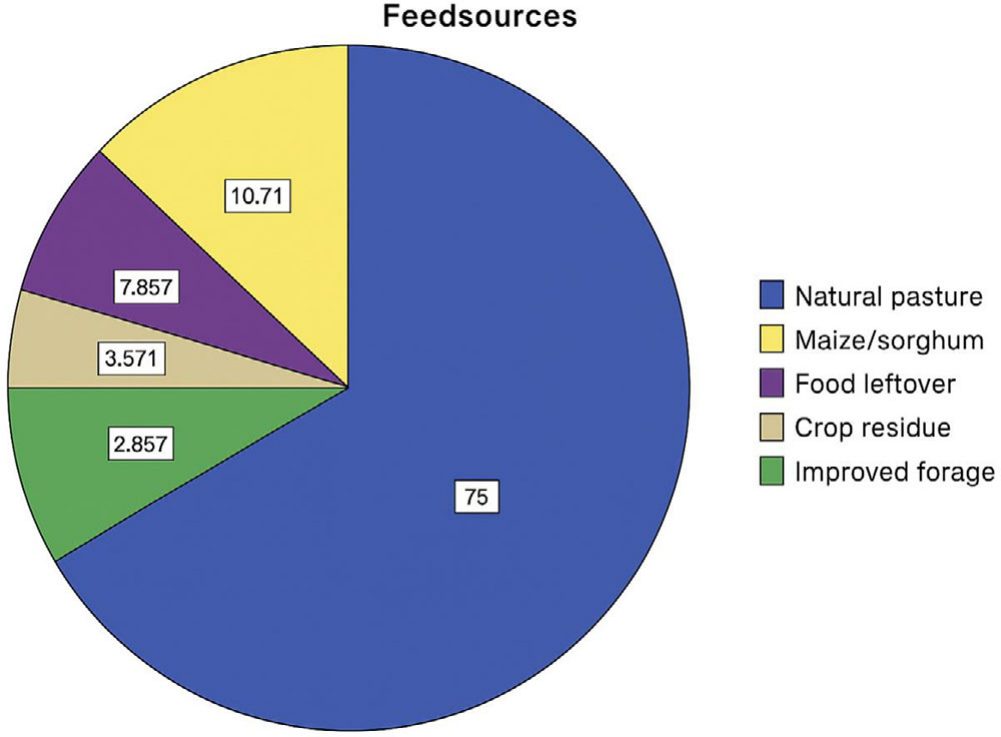
Feed resources available (%HH) in the study areas.

Feed resource availability and nutritional quality are the most important factors that determine livestock productivity. The respondents in the study area indicated that the available feed resources for livestock come from different sources, as presented in Figure 2. The feed resources in the district were natural pasture (75%), crop residues (3.571%), improved forage (2.857%), food leftovers (7.857%) and maize/sorghum (10.71%) (Figure 2). Natural pasture (75%) was the most dominant feed resource for livestock in the study area. No cropland or crop aftermath grazing areas were owned or directly managed by the respondents during the study period. However, some livestock producers utilized crop residues and maize/sorghum obtained through purchase, exchange, or seasonal movement to neighbouring agro‐pastoral and farming areas. As a result, crop residues and cereal grains contributed a small proportion of the available livestock feed resources in the study area.

### 3.4 Feeding System

**FIGURE 3:**
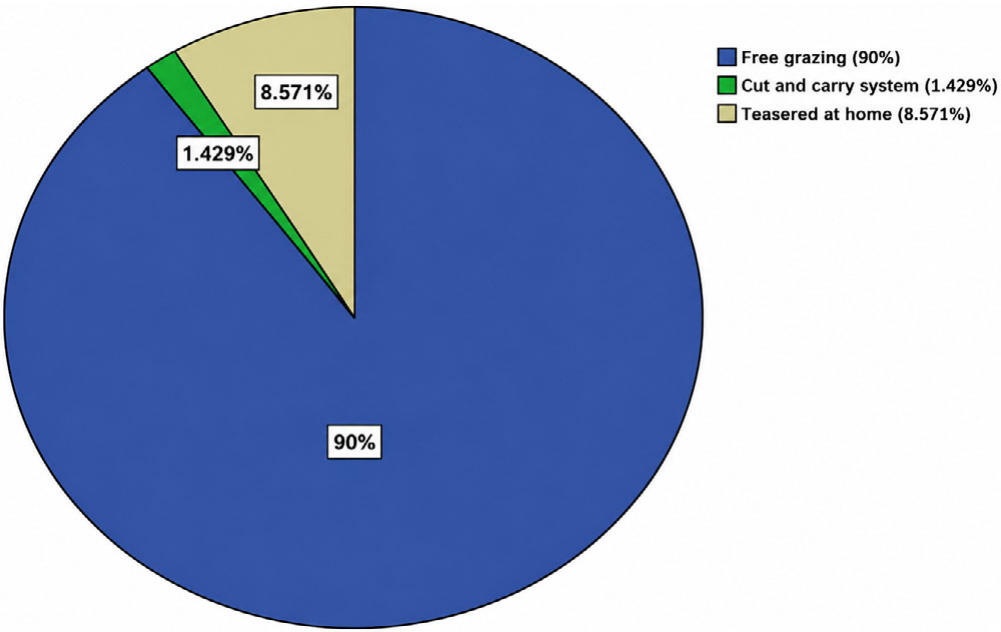
Feeding system (%HH) in the study area.

The feeding system for livestock in the current study area is presented in Figure 3. Free grazing, cut‐and‐carry systems and teasered‐at‐home feed are the existing feeding systems in the selected districts of the Korahey Zone. The majority (90%) of the respondents practiced a natural pasture‐free grazing system, 8.571% teasered at home, and 1.429% cut and carried a feeding system (Figure 3).

### 3.5 Estimation of the DM Requirement and Feed Supply Balance

The mean DM production from grazing land, estimated livestock DM requirements, and feed balance analysis for the study area are presented in Table 2. Feed DM supply from different land‐use types was estimated by multiplying the area under each land‐use category by the corresponding conversion factors. Conversion factors of 2.0, 1.0, 5.0, 1.0 and 1.0 tons DM/ha/year were used for natural pasture, crop residues, improved forages, HH food leftovers (assumed HH contribution), and maize/sorghum by‐products (assumed equivalent), respectively (FAO 1984). Livestock DM requirements were calculated based on the maintenance requirement of a 250‐kg dual‐purpose TLU, which requires 6.25 kg DM per day or 2281 kg (2.281 tons) DM per year per animal (Jahnke 1982).

**TABLE 2:** Estimation of tDM requirements and feed supplies in the study areas.

Feed supply	Area (ha)	DM (ton)
Annual feed DM supply (sampled HH)/dry area	941.6	1883.2
**Feed requirement**		
Total household (HH)		80
Total number of TLU (sampled HH)		1764
Average no. of TLU/HH		22.05
DM required/TLU/year (tons)		2.281
Estimated annual requirement for maintenance		4023.68
Average estimated annual requirement/HH for maintenance		50.29
Balance between feed supply and requirement		−2.140.48
Existing feed supply satisfies (%)		46.80
Proportion of feed gap (%)		53.20

Abbreviations: DM, dry matter; HH, household; tDM, ton dry matter; TLU, tropical livestock unit.

The utilized feed DM supply for the sampled districts was estimated at 2196 tons per year, derived primarily from natural pastureland (Table 2). The total annual livestock DM requirement was estimated at 4023.68 tons. This indicates a negative feed balance of −1827.68 tons DM per year in the study area, demonstrating a substantial deficit between available feed resources and livestock maintenance requirements (Table 2).

### 3.6 Major Feed Resource‐Related Constraints in the Study Area

Drought, grazing land shortages, limited availability of high‐quality forage seeds, inadequate feed quantity and quality, expansion of invasive plant species, insufficient extension services and land‐use conflicts were identified as major feed‐related constraints in the study area (Table 3). Among these, drought was ranked as the most critical constraint, followed by the expansion of invasive species, feed quantity and quality problems, limited access to high‐quality forage seeds and grazing land shortages, which were ranked first to fifth, respectively.

**TABLE 3:** Feed resource‐related constraints in the study area.

Constraints	Woreda							
	Kabridahar		Lass‐Dankhare		Boodele		Shilabo	
	Index	Rank	Index	Rank	Index	Rank	Index	Rank
Drought	0.176383	1	0.174095	1	0.175775	1	0.174157	1
Grazing land shortage	0.139013	5	0.139276	5	0.138848	5	0.139045	5
Lack of high‐quality forage seeds	0.149477	4	0.149025	4	0.149188	4	0.148876	4
Feed quality and quantity problem	0.16293	3	0.16156	3	0.162482	3	0.161517	3
Invasive species expansion	0.170404	2	0.168524	2	0.169867	2	0.168539	2
Lack of extension services	0.103139	6	0.10585	6	0.104874	6	0.106742	6
Land use conflict	0.098655	7	0.101671	7	0.098966	7	0.101124	7

*Note*: Index = Σ of [3 × number of households ranked 1st + 2 × number of households ranked 2nd + 1 × number of households ranked 3rd] given a particular valued feed source divided by Σ of [3 × number of households ranked 1st + 2 × number of households ranked 2nd + 1 × number of households ranked 3rd +….] summed for all valued feed sources.

## 4 Discussion

### 4.1 Livestock Holding and Composition

Livestock population size and species composition varied among the districts, likely reflecting differences in the availability of natural grazing resources. In contrast to the findings of the present study, Endale (2015) reported an average livestock holding of 12.26 TLU per HH in the Meta Robe District of Oromia. Similarly, Tilahun (2022) reported average holdings of 6.04 TLU per HH in mid‐altitude areas and 5.76 TLU per HH in high‐elevation agro‐ecologies of Gondar, both of which are lower than the values observed in the current study. These differences may be attributed to variations in landholding size, agro‐ecological conditions, and the degree to which pastoral HHs rely on livestock for livelihood security. Across all selected districts of the Korahey Zone, camels and goats were the dominant livestock species. Their prevalence can be explained by the suitability of the environment for these species, the availability of browse resources, their high adaptability to hot and arid climates, resilience under drought conditions, the presence of extensive rangelands and the encroachment of grasslands by woody vegetation, which favours browsing species over grazers.

### 4.2 Land Holding Capacity

The larger proportion of landholdings in the study area is allocated to grazing, indicating that pastureland constitutes the principal feed source for livestock in pastoral production systems. This pattern is representative of many pastoral areas of Ethiopia, where livestock production forms the primary livelihood base. The average grazing landholding per HH in the present study area exceeds both the national average landholding size of 0.96 ha per HH (CSA, 2011) and the average grazing landholding of 0.153 ha per HH reported for the Wolaita Zone (Debebe et al. 2021).

These differences in land allocation may be attributed to variations in farming systems, agro‐ecological conditions, human population density, livestock population pressure, landscape characteristics and production objectives. In pastoral areas, greater emphasis is placed on grazing land rather than crop production because livelihoods depend predominantly on livestock. Furthermore, landholdings in lowland areas tend to be relatively large due to lower population density (Getahun and Tegene 2018). In contrast, farmers in highland areas, where mixed crop–livestock systems are practised, allocate a larger proportion of land to crop production and give less emphasis to grazing (Endeshaw 2007; Belete 2009; Abera 2012; Tegene et al. 2016).

### 4.3 Available Feed Resources and Feeding Systems

The high reliance on natural pasture and the limited contribution of crop residues observed in the study area can be attributed to the predominance of pastoral livestock production systems and the availability of extensive grazing lands, in contrast to mixed crop–livestock systems. Natural pasture, particularly shrubs and browse species, serves as a primary feed source for small ruminants (Fikru and Gebeyew 2015; Kebede et al. 2016; Gatew et al. 2017). Asmamaw (2021) similarly reported that natural pasture and browsing species constitute major livestock feed resources, consistent with Teshome (2016), who emphasized their abundance on vast communal grazing lands. Studies conducted in Deghabur (Abshir 2018) and Kebribeyah (Muhyadin 2010) also identified natural pasture as the dominant feed source, reflecting the typical pastoral production system in Ethiopia, where extensive rangeland grazing provides the primary feed for livestock.

Agro‐ecological variation further explains differences in feed resource availability. For example, maize, sorghum and teff straw serve as major feed sources during the dry season in southeastern Ethiopia (Abate et al. 2010), whereas natural pasture dominates both the dry (55.7%) and wet (100%) season feed supply in the Metema District (Tesfaye 2008). In contrast, Duguma et al. (2012) and Melaku (2017) reported that crop residues and crop aftermath grazing constitute the principal feed resources in highland areas. Similarly, natural pasture contributed only about 13% of total feed resources in Bure Woreda (Shitahun 2009) and 25.85% in the Sinana sub‐district (B. Solomon 2004), reflecting greater HH dependence on crop production in those areas.

In the present study, the majority of respondents (90%) practised free grazing on natural pastures. This practice is likely associated with the availability of grazing resources, the dominance of pastoral production systems, minimal crop production, and limited awareness or adoption of tethering and cut‐and‐carry feeding systems. These findings align with Tonamo et al. (2015), Assefa et al. (2015) and Melaku (2017), who reported that free grazing is the dominant feeding system in Ethiopian highlands. Pastoral areas are also characterized by browsing livestock species such as camels and goats. This observation supports Fikru and Gebeyew (2015), who noted that goats primarily depend on browse species; consequently, free grazing remains the principal feeding strategy for goats in the Deghabur District.

### 4.4 Estimation of DM Requirement and Feed Supply Balance

In general, the DM supply in the study area was insufficient to meet the annual livestock DM requirement. However, some feed resources, such as HH food leftovers, sorghum and maize residues, and improved forages were not quantified in the present study due to their limited use, drought conditions and seasonal availability across the surveyed districts. The results indicate a feed gap of 45.4% (equivalent to approximately 166 days of feed inadequacy), meaning that the existing feed supply satisfies only 54.6% (about 199 days) of the livestock maintenance DM requirement in the study area. These findings are consistent with the negative feed balance reported by Tadesse and Solomon (2014) and Kochare et al. (2018). Similarly, Tessema et al. (2003), Bedasa (2012) and Endale (2015) reported that annual DM production was below livestock requirements in the Metema District, the Highlands of the Blue Nile Basin and Meta Robe District, respectively.

Persistent feed deficits have serious consequences, including poor body condition, emaciation, reduced mobility, markedly decreased milk production and increased livestock mortality, affecting millions of animals across pastoral areas, including the Somali Region (FEWS NET 2022). To address these challenges, the study recommends scaling up improved forage production near settlements and irrigated plots, strengthening feed conservation practices through hay and silage making, establishing community feed banks, promoting sustainable woody browse management and safe harvesting practices, adopting community‐based rangeland management (CBRM), facilitating access to agro‐industrial by‐products and market linkages, and implementing supportive policy and institutional measures to reduce the feed gap in the Korahey Zone.

### 4.5 Major Feed Resource‐Related Constraints in the Study Area

The current findings indicate that drought is the primary feed‐related constraint in the study area, ranking above other existing challenges. Reduced rainfall and the frequent recurrence of drought are the main factors limiting both the availability and quality of livestock feed. Drought also severely affects pastoral livelihoods, as livestock and pasture resources often fail to recover, leaving rangelands bare even during periods of rainfall (Abshir 2018). The combination of unpredictable rainfall patterns and ongoing ecological degradation makes drought the most pervasive problem in pastoral areas of Ethiopia. Malede and Takele (2014) further note that one of the most challenging features of Ethiopia's climate is the high interannual variability and erratic nature of rainfall. Following drought, the spread of invasive plant species represents the next most significant challenge for pastoral communities.

## 5 Conclusion and Recommendations

The present study conducted in the Korahey Zone showed that goats, sheep, cattle, camels and poultry are the principal livestock species kept by HHs. Mean herd holdings ranged from 20.45 to 23.95 TLUs per HH, indicating a strong dependence on livestock for livelihoods. Among these species, camels and goats were dominant, reflecting their superior adaptability to the arid and semi‐arid environmental conditions of the area.

Feed resource availability was identified as the primary factor influencing livestock productivity. The major feed sources included natural pasture, crop residues, improved forages, HH food leftovers and maize and sorghum by‐products. Natural pasture was the most important feed resource, contributing approximately 75% of the total feed supply. About 90% of HHs relied on free grazing systems, indicating heavy dependence on communal rangelands.

The estimated annual usable DM production from available feed resources was 2196 tons, whereas the total annual DM requirement for the livestock population was 4023.68 tons. This indicates that the available feed resources satisfy only 54.6% of the maintenance DM requirement, equivalent to approximately 199 days of feed sufficiency per year, demonstrating a substantial negative feed balance in the study area.

Livestock production in the zone is constrained by recurrent drought, shrinking grazing land, limited availability of high‐quality forage seeds, inadequate feed quantity and quality, expansion of invasive plant species, insufficient veterinary and extension services and increasing land‐use conflicts. Addressing these constraints through sustainable rangeland management, introduction of high‐yield and drought‐tolerant forage species, strategic feed supplementation and strengthened veterinary and extension services is essential to improve livestock productivity and support sustainable pastoral and agro‐pastoral livelihoods.

On the basis of the major findings and conclusions of the current study, the following major recommendations are proposed:

- Further studies should be conducted on the availability of all feed resources for more investigations of all available feed resources in the pastoral community.
- The identification of major available feed resources on the basis of seasonal variation should be performed to address problems related to feed shortages.
- A detailed chemical composition analysis should be performed to identify the quality of available feed resources.

## Author Contributions

**Wondimagegn Tadesse**: data collection, formal analysis, writing – original draft, writing – review and editing. **Kibebew Babege**: formal analysis, writing – original draft, writing – review and editing. **Mohamed Abdi**: writing – review and editing.

## Ethics Statement

The authors have nothing to report.

## Consent

The authors have nothing to report.

## Conflicts of Interest

The authors declare no conflicts of interest.

## Data Availability

The data that support the findings of this study are available from the corresponding author upon reasonable request.
